# Association between the life’s essential 8 health behaviors score and all-cause mortality in cardiovascular-kidney-metabolic syndrome patients

**DOI:** 10.3389/fnut.2025.1612693

**Published:** 2025-06-27

**Authors:** Dingyuan Tu, Xiaoli Zuo, Ping Li, Jianming Wang

**Affiliations:** ^1^Department of Congenital Heart Disease, General Hospital of Northern Theater Command, Shenyang, Liaoning, China; ^2^Department of Cardiology, The 961st Hospital of the Joint Logistics Support Force of The Chinese People’s Liberation Army, Qiqihar, Heilongjiang, China

**Keywords:** cardiovascular health, cardiovascular-kidney-metabolic syndrome, health behaviors score, life’s essential 8, mortality, national health and nutrition examination survey

## Abstract

**Background:**

Cardiovascular-kidney-metabolic (CKM) syndrome, a novel and multistage disorder recently proposed by the American Heart Association (AHA), highlights the intricate connection between cardiovascular, renal, and metabolic illnesses. Poor CKM health is highly prevalent in the U.S. We aimed to examine the association of Life’s Essential 8 (LE8), the AHA’s key measures for improving and maintaining cardiovascular health (CVH), with all-cause mortality among U.S. CKM syndrome patients.

**Methods:**

This population-based prospective cohort study analyzed data of adults in the National Health and Nutrition Examination Survey from 2011 to 2018, with linked mortality information until 2019. LE8 score, which included four health behaviors and four health factors, was categorized into low (0–49), moderate (50–79), and high (80–100) CVH. Five CKM syndrome stages (stages 0–4) were defined according to the 2023 AHA guidelines, with advanced (stages 3 or 4) and non-advanced (stages 1 or 2) disease. Multivariable Cox regression and restricted cubic spline models were used to assess the associations.

**Results:**

Among 11,638 CKM syndrome patients (2,060 advanced stages), 831 deaths occurred over a median follow-up of 5.0 years. Interestingly, the relationship between total LE8 score and all-cause mortality was only significant in advanced CKM syndrome patients, while not significant in non-advanced CKM syndrome patients. Further analyses of LE8 subscales revealed that advanced CKM syndrome patients with moderate and high LE8 health behaviors score exhibited a reduced risk of all-cause mortality (moderate: hazard ratio, 0.52; 95% confidence interval, 0.39–0.70; high: 0.35; 0.24–0.49), as well as non-advanced CKM syndrome patients (moderate: 0.75; 0.55–0.98; high: 0.38; 0.24–0.59). Population attributable fraction suggested that 22.5 and 23.9% of all-cause mortality attributed to poor or moderate LE8 health behaviors score could be avoided if high score was achieved in advanced and non-advanced CKM syndrome patients.

**Conclusion:**

Our study revealed a significant association between the LE8 health behaviors score and all-cause mortality in both advanced and non-advanced CKM syndrome patients, underscoring the value of this score in enhancing risk management approaches for CKM syndrome patients in future research and clinical practice.

## Introduction

Cardiovascular disease (CVD) is the leading cause of global mortality and a major contributor to health loss worldwide (1). As heart disease and stroke statistics of 2025 report from American Heart Association (AHA), the prevalence of CVD in U.S. adults age ≥20 years is 48.6% overall and increases with age in both males and females (2). Recently, there is a growing understanding of the science underlying the complex interplay among CVD, chronic kidney disease (CKD), metabolic abnormalities, which presents significant challenges to public health worldwide (3, 4). This poor cardiovascular-kidney metabolic (CKM) health has profound implications for clinical outcomes, mainly by increasing the risks of cardiovascular problems and early mortality (5, 6). Consequently, there is an urgent demand for new approaches to tackle risk factors and alleviate the burden of CVD.

In the Presidential Advisory issued by the AHA in October 2023, CKM syndrome was introduced and characterized as a systemic disorder of metabolic disorders, alongside kidney diseases and cardiovascular conditions (7). The CKM syndrome reflects the complex interactions among obesity, diabetes, CKD, CVD, and is classified into five stages based on the presence of CKM risk factors, ranging from stage 0 (no risk factors present) to stage 4 (clinical CVD). Recent research discovered that CKM syndrome affects a large portion of adults in the U.S, with 90% having CKM syndrome (stage 1 or higher), and 15% diagnosed with advanced stages (stage 3 or 4) (8). Besides, higher CKM syndrome stage was associated with a higher risk of all-cause mortality (6). Thus, efforts to better manage CKM syndrome patients and reduce mortality are highly warranted.

Modifiable risk factors like elevated body mass index (BMI), tobacco use, unhealthy diet, physical inactivity, account for a proportion of prevalent and incident CVD (9). To reduce the burden of CVD, in 2022 the AHA developed and updated the concept of cardiovascular health (CVH), which comprises four health behaviors (diet, physical activity, nicotine exposure, and sleep duration) and four health factors (BMI, non-high-density lipoprotein [non-HDL] cholesterol, blood glucose, and blood pressure) (10). This new CVH construct, termed Life’s Essential 8 (LE8), was scored from 0 to 100 to better quantify individual differences in CVH. The LE8 score has demonstrated inverse associations with adverse outcomes, such as CVD (11), CKD (12), diabetes (13), and all-cause and CVD mortality (14). Given the correlation between LE8 score and components of CKM syndrome, we conjecture that a higher LE8 score was associated with a reduced risk of all-cause mortality in CKM syndrome patients.

In our study, we utilized data from the National Health and Nutrition Examination Survey (NHANES) to investigate the association of the LE8 score with all-cause mortality among U.S. adults. In addition, we also calculated population attributable fraction (PAF) of LE8 score in relation to mortality risk.

## Methods

### Study design and participants

The NHANES is a series of ongoing cross-sectional surveys conducted by the National Center for Health Statistics at the Centers for Disease Control and Prevention. It provides nationally representative cross-sectional data for the health status of the U.S. civilian noninstitutionalized population. Participants were interviewed at home regarding demographic, socioeconomic, dietary, and health-related questions. Subsequently, they underwent medical, dental, and physiological measurements as well as laboratory tests at a mobile examination center. Ethical approval for the study was obtained from the Research Ethics Review Board of National Center for Health Statistics, and all participants gave written informed consent. This study adhered to the Strengthening the Reporting of Observational Studies in Epidemiology (STROBE) reporting guideline.

**Figure 1:**
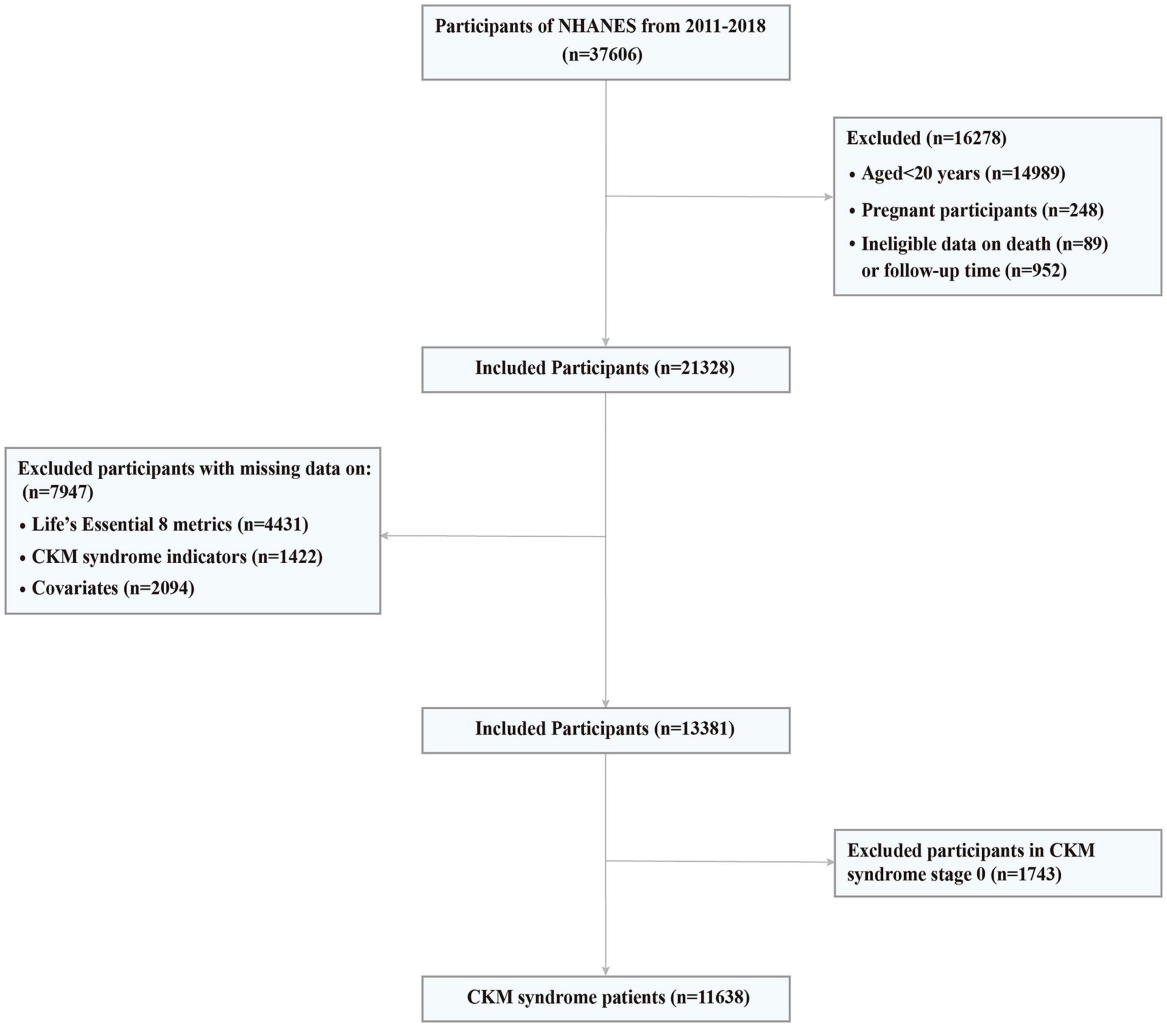
Screening flow of participants. CKM, Cardiovascular-Kidney-Metabolic; NHANES, National Health and Nutrition Examination Survey.

The continuous NHANES began in 1999 and collects data biennially. In CKM syndrome stage 0 and 1, lower BMI and waist circumference cut point is advised for Asian populations given their predisposition to developing metabolic abnormalities at lower levels of adiposity (15). Since NHANES collected responses on Asian race as a separate category from the 2011–2012 cycle, we used data from four NHANES cycles, including NHANES 2011–2012, NHANES 2013–2014, NHANES 2015–2016, and NHANES 2017–2018. The link to the dataset can be found in the “Data availability” section. A total of 22,617 adults aged 20 years or older were included. We further excluded participants having the following conditions: (1) a pregnancy at baseline (*n* = 248); (2) ineligible data on death or follow-up (*n* = 1,041); (3) missing LE8 CVH scores (*n* = 4,431); (4) missing information on CKM syndrome indicators (*n* = 1,422); (5) missing information on potential covariates (*n* = 2,094). Consequently, the final analytical sample included in this study consisted of 13,381 adults (Figure 1).

### Measurement of LE8

The LE8 score comprises four health behaviors (diet, physical activity, nicotine exposure, and sleep duration) and four health factors (BMI, non-HDL cholesterol, blood glucose, and blood pressure), each with its own scoring standards ranging from 0 to 100 (10). Dietary intake was obtained by two non-consecutive 24-h dietary recalls, and the dietary quality was assessed by Healthy Eating Index 2015 (HEI-2015) scores, which aligns with the 2020–2025 Dietary Guidelines for Americans (16). Information on physical activity (self-reported minutes per week of moderate-to-vigorous physical activity), nicotine exposure (combustible cigarette use, inhaled nicotine delivery systems use, and secondhand smoke exposure) sleeping information (sleep duration), and medication use were obtained from standardized questionnaires. Height, weight, and systolic and diastolic blood pressure were measured at the physical examination. BMI was calculated as weight in kilograms divided by height in meters squared. Blood lipid and glycemic profiles were measured at a morning examination session after fasting for 9 h or more. The non-HDL cholesterol was calculated by total cholesterol minus high-density lipoprotein (HDL) cholesterol, and Hemoglobin A1c (HbA1c) was utilized to evaluate blood glucose levels. Detailed algorithms for calculating the LE8 scores for each of the eight metrics to NHANES data are provided in Supplementary Table S1. In brief, the overall LE8 score was calculated as the average of the eight CVH metrics. The overall CVH score was further categorized into low (0–49 points), moderate (50–79 points), and high (80–100 points) CVH groups, following the recommendations of AHA (10). Additionally, we used the same criteria and cut-off points to measure and categorize LE8 health behaviors and factors score to further investigate the association between LE8 subscales and all-cause mortality in patients with CKM syndrome. These definitions are consistent with previous studies (17, 18).

### Definitions of the components of CKM syndrome

The AHA criteria define CKM syndrome stages based on ten components: BMI, waist circumference, hypertension, prediabetes, diabetes, hypertriglyceridemia, metabolic syndrome, CKD, 10-year CVD risk, and clinical CVD (7). Hypertension was characterized by systolic blood pressure ≥ 140 mmHg, diastolic blood pressure≥90 mmHg, a self-reported history of high blood pressure, or antihypertensive drug intake. Prediabetes was defined as having a fasting blood glucose level from 100 to 124 mg/dL, or a HbA1c of 5.7 to 6.4%. Diabetes was identified by a fasting blood glucose ≥ 125 mg/dL, a HbA1c ≥ 6.5%, self-reported history of diabetes, or current hypoglycemic treatment. Metabolic syndrome was defined as presence of any 3 of the following 5 elements: (1) the fasting blood glucose>100 mg/dL or drug treatment for diabetes mellitus; (2) HDL cholesterol <50 mg/dL in females, <40 mg/dL in males or drug treatment for reduced HDL cholesterol; (3) plasma triglyceride >150 mg/dL or drug treatment for raised triglyceride; (4) waist circumference > 88 cm in women or >102 cm in men; (5) blood pressure > 130/85 mmHg or drug treatment for raised blood pressure. Based on the Kidney Disease Improving Global Outcome criteria, CKD status was categorized as: low risk, moderate risk, high risk, and very high risk (Supplementary Table S2) (7). 10-year CVD risk was estimated with the AHA Predicting Risk of CVD EVENTs (PREVENT) equations (19).

### Definition of CKM syndrome stages

In the present study, the definition of CKM syndrome stage was determined according to the AHA Presidential Advisory Statement on CKM syndrome (7), with modifications for NHANES data based on the criteria from Aggarwal et al. (8). Stage 0: Individuals with normal BMI and waist circumference, normoglycemia, normotension, normal lipid status, and no evidence of CKD or subclinical or clinical CVD. Stage 1: Individuals having excess weight (BMI ≥ 25 kg/m2 or ≥23 kg/m2 if Asian ethnicity), or abdominal obesity (waist circumference≥88/102 cm in women/men or 80/90 cm in women/men if Asian ethnicity), or prediabetes. Stage 2: Individuals with metabolic risk factors (hypertriglyceridemia [≥135 mg/dL], hypertension, metabolic syndrome, diabetes), or moderate-to-high-risk CKD. Stage 3: Individuals at very high risk of CKD or a high risk (≥20%) of 10-year CVD. Stage 4: Individuals with a self-reported diagnosis of CVD (coronary heart disease, angina, heart attack, heart failure, or stroke). The detailed descriptions of CKM syndrome stage definitions are in Supplementary Table S3. Based on the prevalence of CKM syndrome among U.S. adults (8), CKM syndrome included stage 1 or higher, with advanced stages being stage 3 or 4, as these indicated participants with or at high risk for CVD.

### Ascertainment of mortality

Data for deaths were obtained by linking to the NHANES-linked National Death Index until December 31, 2019 (20). Cause of death was defined using the International Statistical Classification of Disease, Tenth Revision (ICD-10). Death from all reasons was defined as all-cause mortality. Follow-up time was calculated from the date of interview to the date of death, or the end of follow-up (December 31, 2019), whichever came first.

### Definition of covariates

In line with previous studies (14, 18, 21, 22), the following covariates were included the current study: age, sex (female or male), ethnicity (non-Hispanic White, non-Hispanic Black, non-Hispanic Asian, Hispanic and other race or ethnicity), education level (less than high school, high school or equivalent, and college or above), ratio of family income to poverty (less than 1, 1 to 3, and more than 3), marital status (coupled, and single or separated), BMI, HbA1c, total cholesterol, HDL cholesterol, low-density lipoprotein (LDL) cholesterol, estimated glomerular filtration rate (eGFR), alcohol consumption status (non-drinker, low to moderate drinker, and heavy drinker), history of cancer (yes or no), and the use of antihypertensive, antidiabetic, lipid-lowering medications.

### Statistical analysis

All analyses were performed incorporating the sampling weights to account for the complex NHANES sampling design. For baseline characterization, continuous variables were described by weighted mean value with weighted standard errors (SE), while categorical variables were expressed as number with weighted percentage (%). To check for differences in characteristics between the three CVH groups, Analysis of Variance was used for differences in weighted means for continuous variables and the Rao-Scott Chi-Square test for differences in weighted percentages for categorical variables. Survey-weighted Cox proportional hazards models were performed to estimate hazard ratios (HR) and 95% confidence intervals (CI) for the associations of LE8 and LE8 subscales with all-cause mortality. We fitted two statistical models. Model 1 was adjusted for sociodemographic variables, including age, sex, ethnicity, educational level, family income, and marital status. Model 2 was adjusted for the variables in Model 1 and additional confounders including BMI, HbA1c, total cholesterol, HDL cholesterol, LDL cholesterol, eGFR, alcohol status, history of cancer, and the use of antihypertensive, antidiabetic, lipid-lowering medications. The Schoenfeld residuals test is used to test the proportional hazard assumption in Cox model.

To comprehensively understand the relationship between LE8 and mortality risk, restricted cubic spline (RCS) models were utilized to estimate the dose–response association of total LE8 score, LE8 health behaviors score, and LE8 health factors score with all-cause mortality. We used the Akaike information criterion (AIC) to select the RCS model with specific number of knots and adopted the model with the lowest AIC criterion value, which is considered as the best-fitting RCS model. Besides, the PAF was calculated to quantify the proportion of mortality in a population attributable to exposure to a risk factor (23). In our study, the ‘graphPAF’ R package was used to calculate the adjusted PAF of high CVH score (≥80 points) vs. moderate or low CVH score (<80 points) to quantify the proportion of all-cause mortality that could be prevented by improving CVH score.

Subgroup analyses were performed to examine the association of LE8 score with all-cause mortality stratified by age, sex, ethnicity, education level, family income, marital status, alcohol status, cancer, and the use of antihypertensive, antidiabetic, lipid-lowering medications. Sensitivity analyses were performed to assess the robustness of the results. First, we excluded participants who experienced mortality during the first year of follow-up to account for reverse causality. Second, individuals with a self-reported history of cancer were excluded. Third, iterative imputation for missing covariates was performed using a machine learning algorithm via the R package ‘missRanger’ to test the above associations. The missRanger is an iterative imputation approach based on random forest (24). Missing values for a variable are imputed using predictions made by a random forest, which employs all remaining variables as covariates. The algorithm performs repeated iterations across all variables. This process continues until there is no further improvement observed in the average Out-Of-Bag prediction error of the models, which serves as the stopping criterion for the iterations. Besides, the missRanger incorporates in the imputation process the predictive mean matching option, which is employed to avoid imputation with values not present in the original data. We employed the parameters (pmm.k = 3, num.trees = 500) in the imputation process.

All analyses were conducted using R software version 4.4.2. A two-tailed *p* value < 0.05 was considered statistically significant.

## Results

### Basic characteristics

Of the 13,381 U.S. adults from NHANES 2011 to 2018, the proportion of those in stage 0 to 4 was 13.02, 26.84, 44.74, 4.54 and 10.86%, respectively. 86.98% of U.S. adults met criteria for CKM syndrome (stage 1 or higher) and 15.40% met criteria for advanced stages. We then removed 1,743 participants in CKM syndrome stage 0, and finally this study included 11,638 CKM syndrome patients (Figure 1).

The baseline characteristics of the CKM syndrome patients were summarized by the three categories of LE8 score in Table 1. Only 14.63% of CKM syndrome patients achieved a high LE8 score, whereas 15.04% had a low score. CKM syndrome patients with higher LE8 score were more likely to be younger, female, non-Hispanic White and Non-Hispanic Asian, coupled, non-heavy drinkers, non-cancer population, and have a higher education level, higher household income, and higher eGFR. Besides, there were lower proportions of the use of antihypertensive drugs, antidiabetic drugs, and antihyperlipidemic drugs in the higher LE8 score group. For LE8 components, participants with a high LE8 score exhibited characteristics of lower BMI, non-HDL cholesterol, HbA1c, blood pressure level, and a higher HEI-2015 score, as well as longer physical activity and sleep duration (all *p* < 0.0001). Besides, CKM syndrome patients in advanced stages were less likely to have high LE8 scores (all *p* < 0.0001).

**Table 1:** Baseline characteristics of CKM syndrome patients by different levels of CVH estimated by the LE8 score.

Characteristic	LE8 score			*p-*value
	0–49	50–79	80–100	
No. of participants	1750	8,185	1703	
Prevalence, %	15.04	70.33	14.63	
Age (years), mean (SE)	54.75(0.50)	50.59(0.34)	44.87(0.63)	<0.0001
Sex, *n* (%)				0.002
Female	919(53.01)	4,037(49.25)	968(56.12)	
Male	831(46.99)	4,148(50.75)	735(43.88)	
Race or ethnicity, *n* (%)				<0.0001
Hispanic	382(12.40)	1962(13.76)	375(13.90)	
Non-Hispanic Asian	40(1.04)	723(3.57)	384(8.48)	
Non-Hispanic Black	515(15.14)	1842(10.52)	220(5.83)	
Non-Hispanic White	728(66.70)	3,376(69.03)	678(68.99)	
Other	85(4.72)	282(3.13)	46(2.80)	
Education level, *n* (%)				<0.0001
Less than high school	508(21.97)	1,576(12.42)	183(6.16)	
High school or equivalent	482(30.16)	1949(24.51)	234(13.25)	
College or above	760(47.88)	4,660(63.07)	1,286(80.58)	
Family PIR, *n* (%)				<0.0001
<1.0	528(22.27)	1,562(12.28)	256(9.89)	
1.0–3.0	840(44.22)	3,544(37.25)	549(26.26)	
>3.0	382(33.51)	3,079(50.47)	898(63.85)	
Marital status, *n* (%)				<0.001
Coupled	942(59.86)	4,967(64.85)	1,113(69.46)	
Single or separated	808(40.14)	3,218(35.15)	590(30.54)	
Alcohol status, *n* (%)				<0.0001
Non-drinker	607(29.40)	2,310(22.31)	417(18.60)	
Low to moderate drinker	784(49.65)	4,297(56.63)	1,046(65.83)	
Heavy drinker	359(20.95)	1,578(21.05)	240(15.57)	
History of cancer, *n* (%)				<0.001
Yes	195(13.07)	908(12.27)	120(8.40)	
No	1,555(86.93)	7,277(87.73)	1,583(91.60)	
eGFR (ml/min/1.73m^2^), mean (SE)	87.14(0.75)	91.52(0.44)	95.54(0.81)	<0.0001
Antihypertensive drugs, n (%)				<0.0001
Yes	1,054(55.30)	3,112(35.13)	238(12.34)	
No	696(44.70)	5,073(64.87)	1,465(87.66)	
Antidiabetic drugs, *n* (%)				<0.0001
Yes	588(30.49)	1,063(10.42)	29(1.10)	
No	1,162(69.51)	7,122(89.58)	1,674(98.90)	
Antihyperlipidemic drugs, *n* (%)				<0.0001
Yes	666(36.24)	2010(23.44)	201(12.45)	
No	1,084(63.76)	6,175(76.56)	1,502(87.55)	
Diet				<0.0001
Mean score, mean (SE)	20.82(0.72)	35.88(0.66)	62.04(1.05)	
HEI-2015 score	42.79(0.34)	49.58(0.30)	61.09(0.55)	
Physical activity				<0.0001
Mean score, mean (SE)	28.49(1.74)	74.24(0.70)	96.86(0.33)	
Minutes per week	523.42(32.10)	1004.47(29.79)	1052.57(32.96)	
Nicotine exposure				<0.0001
Mean score, mean (SE)	43.89(1.41)	73.12(0.70)	92.42(0.58)	
Sleep health				<0.0001
Mean score, mean (SE)	67.87(1.03)	84.14(0.37)	93.35(0.51)	
Hours per day	6.54(0.05)	7.12(0.02)	7.44(0.03)	
Body mass index				<0.0001
Mean score, mean (SE)	28.05(0.96)	49.47(0.56)	74.21(0.72)	
kg/m2	35.91(0.27)	30.88(0.12)	26.57(0.12)	
Blood lipids				<0.0001
Mean score, mean (SE)	44.20(1.02)	61.18(0.55)	81.81(0.88)	
Non-HDL cholesterol, mg/dL	162.40(1.61)	143.36(0.87)	121.18(1.06)	
Blood glucose				<0.0001
Mean score, mean (SE)	57.32(0.89)	82.69(0.37)	96.68(0.37)	
HbA1c, %	6.51(0.04)	5.68(0.01)	5.29(0.01)	
Blood pressure				<0.0001
Mean score, mean (SE)	44.23(0.93)	63.47(0.50)	86.65(0.70)	
Systolic, mmHg	133.53(0.67)	124.71(0.26)	114.47(0.47)	
Diastolic, mmHg	73.74(0.56)	72.03(0.25)	68.64(0.40)	
CKM syndrome stage, n (%)				<0.0001
Stage 1	165(10.58)	2,444(33.11)	982(58.98)	
Stage 2	1,002(53.83)	4,374(51.11)	611(34.79)	
Stage 3	167(13.48)	410(6.11)	30(1.78)	
Stage 4	416(22.11)	957(9.67)	80(4.45)	
Advanced CKM syndrome, *n* (%)				<0.0001
Yes	583(35.59)	1,367(15.78)	110(6.23)	
No	1,167(64.41)	6,818(84.22)	1,593(93.77)	

Values are weighted mean (SE) for continuous variables or numbers (weighted %) for categorical variables. Because all numbers were rounded, percentages may not total 100%. CVH, cardiovascular health; eGFR, estimated glomerular filtration rate; HbA1c, Hemoglobin A1c; HEI-2015, Healthy Eating Index 2015; LE8, Life’s Essential 8; non-HDL, non-high-density lipoprotein; PIR, poverty income ratio.

### Association of total LE8 score with all-cause mortality

**Figure 2:**
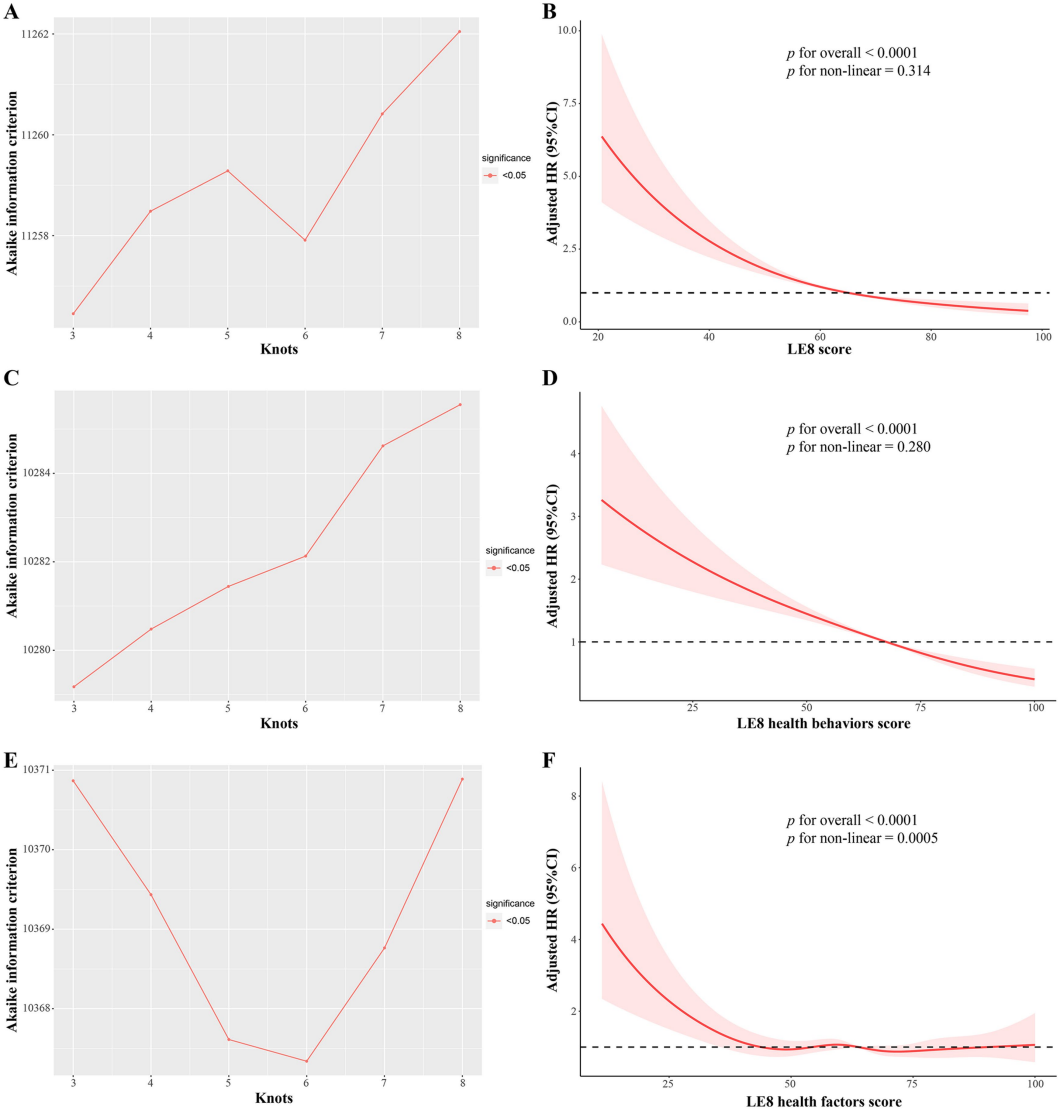
The multivariable adjusted RCS models for association of LE8 score and its subscales with all-cause mortality in CKM syndrome patients. **(A,B)** Total LE8 score; **(C,D)** LE8 health behaviors score; **(E,F)** LE8 health factors score. **(A)** The RCS model with 3 knots showed the lowest AIC statistics; **(B)** Association of total LE8 score with all-cause mortality in CKM syndrome patients; **(C)** The RCS model with 3 knots showed the lowest AIC statistics; **(D)** Association of LE8 health behaviors score with all-cause mortality in CKM syndrome patients; **(E)** The RCS model with 6 knots showed the lowest AIC statistics; **(F)** Association of LE8 health factors score with all-cause mortality in CKM syndrome patients. AIC, Akaike information criterion; CKM, Cardiovascular-Kidney-Metabolic; LE8, Life’s Essential 8; RCS, restricted cubic spline.

During a median follow-up of 5.0 years, a total of 831 deaths were identified. The Schoenfeld residuals suggested that the Cox models met proportional hazards assumptions (all P for global Schoenfeld test > 0.05) (Supplementary Table S4). In the RCS analysis of total LE8 score and all-cause mortality, the use of AIC statistics indicated that the RCS function with 3 knots was the optimal model (lowest AIC: 11256.45) (Figure 2A and Supplementary Table S5). The RCS model revealed that within the context of the fully adjusted model (Model 2), there were approximately a negative linear dose–response association between increased total LE8 score and a reduced risk of all-cause mortality in CKM syndrome patients (*p* for non-linear > 0.05, Figure 2B). Compared to CKM syndrome patients in the low total LE8 CVH group, the moderate and high total LE8 CVH groups was associated with a 48% (HR, 0.52; 95% CI, 0.42–0.64; *p* < 0.0001) and 70% (HR, 0.30; 95% CI, 0.20–0.46; *p* < 0.0001) lower risk of all-cause mortality, respectively. HR for every 10 scores increase in total LE8 score was 0.68 (95% CI, 0.64–0.74; *p* < 0.0001) in association with all-cause mortality (Table 2).

**Table 2:** Association between total LE8 score and all-cause mortality in CKM syndrome patients.

Total LE8 score	Model 1		Model 2	
	HR (95% CI)	*p-*value	HR (95% CI)	*p-*value
Overall				
Low (0–49)	1 (reference)		1 (reference)	
Moderate (50–79)	0.57(0.47,0.70)	<0.0001	0.52(0.42,0.64)	<0.0001
High (80–100)	0.36(0.22,0.56)	<0.0001	0.30(0.20,0.46)	<0.0001
Per 10 score increase	0.76(0.71,0.81)	<0.0001	0.68(0.64,0.74)	<0.0001
Advanced CKM syndrome				
Low (0–49)	1 (reference)		1 (reference)	
Moderate (50–79)	0.51(0.40,0.65)	<0.0001	0.51(0.37,0.69)	<0.0001
High (80–100)	0.31(0.15,0.61)	<0.001	0.35(0.17,0.72)	0.005
Per 10 score increase	0.72(0.65,0.79)	<0.0001	0.67(0.59,0.76)	<0.0001
Non-advanced CKM syndrome				
Low (0–49)	1 (reference)		1 (reference)	
Moderate (50–79)	0.81(0.60,1.10)	0.18	0.81(0.60,1.11)	0.19
High (80–100)	0.55(0.26,1.13)	0.10	0.56(0.27,1.16)	0.12
Per 10 score increase	0.85(0.77,0.94)	0.001	0.73(0.64,0.83)	<0.0001

Model 1: adjusted for sociodemographic variables (age, sex, ethnicity, educational level, family income, and marital status). Model 2: Model 1 + body mass index, hemoglobin A1c, total cholesterol, high-density lipoprotein cholesterol, low-density lipoprotein cholesterol, estimated glomerular filtration rate, alcohol status, history of cancer, and the use of antihypertensive, antidiabetic, lipid-lowering medications. CI, confidence intervals; CKM, cardiovascular-kidney-metabolic; HR, hazard ratio; LE8, Life’s Essential 8.

When stratified by advanced stages, interestingly, a reduced risk of all-cause mortality was observed only among advanced CKM syndrome patients within the moderate and high total LE8 CVH groups when compared to all-cause mortality risk in the low total LE8 CVH group (HR, 0.51; 95% CI, 0.37–0.69; *p* < 0.0001 and HR, 0.35; 95% CI, 0.17–0.72; *p* = 0.005, respectively). However, this association was not observed among non-advanced CKM syndrome patients (HR, 0.81; 95% CI, 0.60–1.11; *p* = 0.19 and HR, 0.56; 95% CI, 0.27–1.16; *p* = 0.12, respectively). These findings suggest that the total LE8 CVH score may not be suitable for determining the risk of all-cause mortality in non-advanced CKM syndrome patients.

### Association of LE8 health behaviors and factors score with all-cause mortality

Given that the total LE8 CVH score contains aspects that partially overlap with the definitions of the components of CKM syndrome, such as BMI, blood lipid profile, blood glucose, and blood pressure, its effect on CKM syndrome might be lessened. Therefore, we divided the total LE8 score into two categories: health behaviors score (including diet, physical activity, nicotine exposure, and sleep duration) and health factors score (including BMI, non-HDL, blood glucose, and blood pressure). The RCS model with 3 knots (lowest AIC: 10279.18, Figure 2C and Supplementary Table S6) revealed that there were approximately a negative linear dose–response association between increased health behaviors score and a reduced risk of all-cause mortality in CKM syndrome patients (*p* for non-linear > 0.05, Figure 2D). Conversely, the RCS model with 6 knots (lowest AIC: 10367.34, Figure 2E and Supplementary Table S7) showed that the association of health factors score with all-cause mortality was nonlinear (*p* < 0.0001) and displayed a W-shaped pattern (Figure 2F). In comparison to their counterparts in the low health behaviors score group, CKM syndrome patients in the moderate and high health behaviors score groups still exhibited a significant decrease in HRs for all-cause mortality, as well as advanced and non-advanced CKM syndrome patients (Table 3). However, this significance was not observed within the health factors score category (Table 4).

**Table 3:** Association between LE8 health behaviors score and all-cause mortality in CKM syndrome patients.

Health behaviors score	Model 1		Model 2	
	HR (95% CI)	*p-*value	HR (95% CI)	*p-*value
Overall				
Low (0–49)	1 (reference)		1 (reference)	
Moderate (50–79)	0.58(0.49,0.70)	<0.0001	0.59(0.49,0.71)	<0.0001
High (80–100)	0.32(0.24,0.42)	<0.0001	0.33(0.25,0.44)	<0.0001
Per 10 score increase	0.80(0.77,0.83)	<0.0001	0.81(0.78,0.84)	<0.0001
Advanced CKM syndrome				
Low (0–49)	1 (reference)		1 (reference)	
Moderate (50–79)	0.51(0.39,0.68)	<0.0001	0.52(0.39,0.70)	<0.0001
High (80–100)	0.32(0.22,0.47)	<0.0001	0.35(0.24,0.49)	<0.0001
Per 10 score increase	0.78(0.73,0.83)	<0.0001	0.78(0.73,0.83)	<0.0001
Non-advanced CKM syndrome				
Low (0–49)	1 (reference)		1 (reference)	
Moderate (50–79)	0.75(0.56,0.97)	0.04	0.75(0.55,0.98)	0.04
High (80–100)	0.38(0.24,0.59)	<0.0001	0.38(0.24,0.59)	<0.0001
Per 10 score increase	0.85(0.79,0.91)	<0.0001	0.84(0.79,0.90)	<0.0001

Model 1: adjusted for sociodemographic variables (age, sex, ethnicity, educational level, family income, and marital status). Model 2: Model 1 + body mass index, hemoglobin A1c, total cholesterol, high-density lipoprotein cholesterol, low-density lipoprotein cholesterol, estimated glomerular filtration rate, alcohol status, history of cancer, and the use of antihypertensive, antidiabetic, lipid-lowering medications. CI, confidence intervals; CKM, cardiovascular-kidney-metabolic; HR, hazard ratio; LE8, Life’s Essential 8.

**Table 4:** Association between LE8 health factors score and all-cause mortality in CKM syndrome patients.

**Health factors score**	Model 1		Model 2	
	HR (95% CI)	*p-*value	HR (95% CI)	*p-*value
Overall				
Low (0–49)	1 (reference)		1 (reference)	
Moderate (50–79)	0.78(0.64,0.94)	0.01	0.90(0.71,1.13)	0.36
High (80–100)	0.88(0.69,1.13)	0.33	0.97(0.67,1.41)	0.88
Per 10 score increase	0.94(0.89,1.01)	0.07	0.93(0.85,1.02)	0.11
Advanced CKM syndrome				
Low (0–49)	1 (reference)		1 (reference)	
Moderate (50–79)	0.80(0.62,1.02)	0.07	1.15(0.87,1.53)	0.33
High (80–100)	0.79(0.48,1.30)	0.35	1.37(0.69,2.73)	0.37
Per 10 score increase	0.91(0.83,1.01)	0.06	1.01(0.88,1.15)	0.90
Non-advanced CKM syndrome				
Low (0–49)	1 (reference)		1 (reference)	
Moderate (50–79)	0.94(0.65,1.35)	0.74	0.72(0.45,1.14)	0.16
High (80–100)	1.17(0.76,1.78)	0.48	0.67(0.36,1.25)	0.21
Per 10 score increase	1.03(0.94,1.13)	0.53	0.88(0.74,1.03)	0.11

Model 1: adjusted for sociodemographic variables (age, sex, ethnicity, educational level, family income, and marital status). Model 2: Model 1 + body mass index, hemoglobin A1c, total cholesterol, high-density lipoprotein cholesterol, low-density lipoprotein cholesterol, estimated glomerular filtration rate, alcohol status, history of cancer, and the use of antihypertensive, antidiabetic, lipid-lowering medications. Abbreviations: CI, confidence intervals; CKM, cardiovascular-kidney-metabolic; HR, hazard ratio; LE8, Life’s Essential 8.

### PAF of LE8 to all-cause mortality

**Figure 3:**
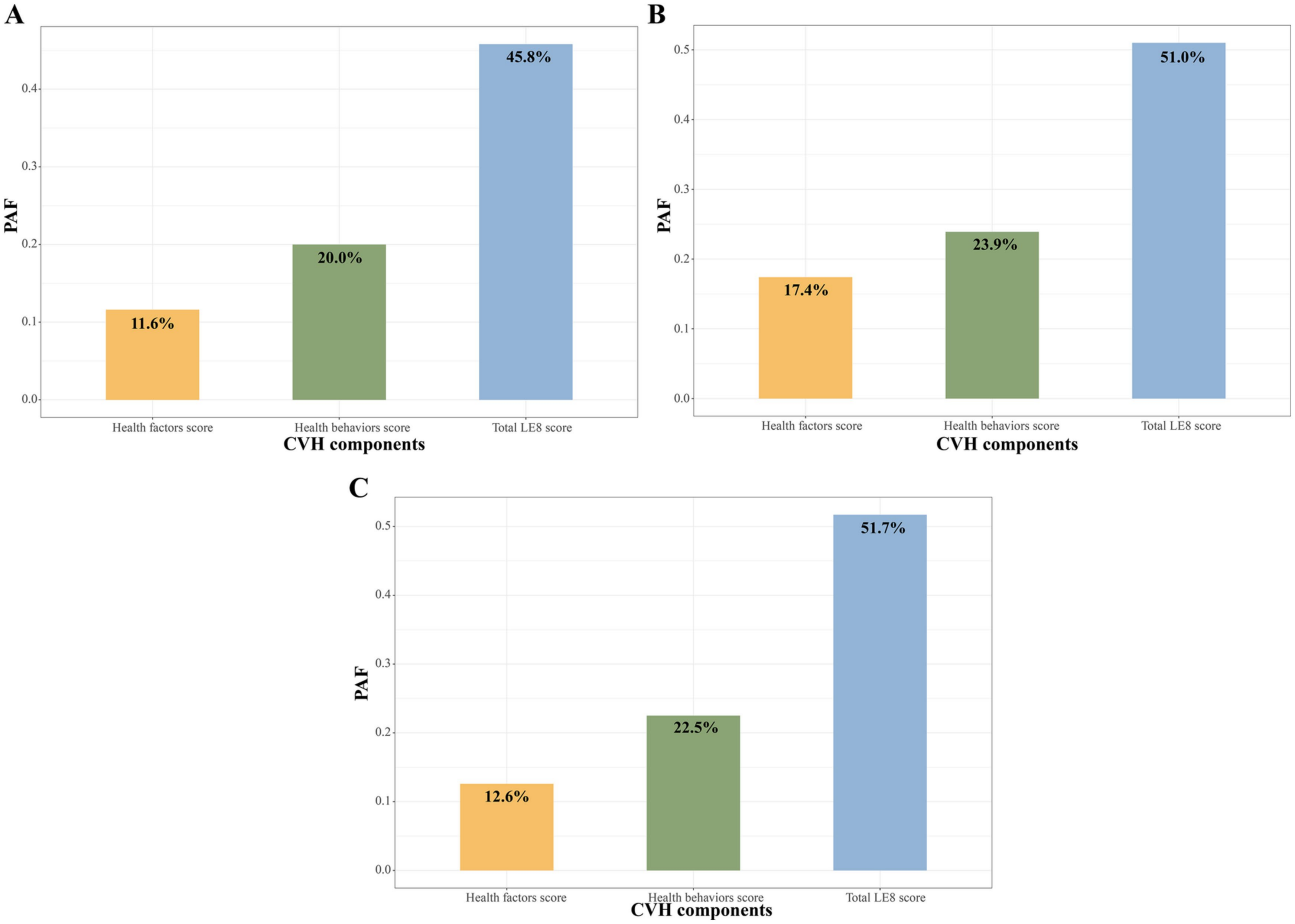
PAF of LE8 score to all-cause mortality among CKM syndrome patients **(A)**, non-advanced **(B)** and advanced **(C)** CKM syndrome patients. CKM, Cardiovascular-Kidney-Metabolic; CVH, Cardiovascular health; LE8, Life’s Essential 8; PAF, population attributable fraction.

Figure 3A shows that the adjusted PAF of high (≥80 points) vs. moderate or low CVH score (<80 points) with all-cause mortality in CKM syndrome patients, and the three metrics were ranked in the relative order of higher to lower fractions as follows: total LE8 score (45.8%), health behaviors score (20.0%), health factors score (11.6%). For advanced and non-advanced CKM syndrome patients, the order of PAFs remained consistent (Figures 3B,C).

### Subgroup and sensitivity analyses

Subgroup analyses by age, sex, ethnicity, education level, family income, marital status, alcohol status, cancer and the use of antihypertensive, antidiabetic, lipid-lowering medications demonstrated similar associations between LE8 health behaviors score and all-cause mortality in the CKM syndrome group (Supplementary Table S8), as well as advanced and non-advanced CKM syndrome patients (Supplementary Tables S9, S10). Besides, the patterns of moderate and high health behaviors score in relation to reduced risk of all-cause mortality remained consistent after excluding participants death within the first year of follow-up (Supplementary Table S11) or with cancer history (Supplementary Table S12), as well as iterative imputation for missing covariates (Supplementary Table S13).

## Discussion

In this large, prospective cohort study of U.S. adults with CKM syndrome, we discovered that the LE8 health behaviors score, rather than the total LE8 score, was significantly correlated with all-cause mortality in both advanced and non-advanced CKM syndrome patients. Our adjusted PAF estimates suggested that 22.5 and 23.9% of all-cause mortality associated with low or moderate health behaviors score could be avoided/eliminated if high health behaviors score were achieved in advanced and non-advanced CKM syndrome patients, respectively. These results highlight the significance of maintaining healthy lifestyle behaviors in CKM syndrome patients.

Non-communicable diseases (NCDs), which primarily include CVD, CKD, chronic respiratory diseases, cancers and diabetes, are the leading cause of morbidity and mortality worldwide. The Global Burden of Disease Study reveals that the proportion of deaths caused by NCDs increased from 56.8% in 1990 to 74.4% in 2019 (25). Additionally, their reference forecast predicts that there will be a continued shift in disease burden to NCDs between 2022 and 2050 (26). CVD accounts for most deaths from NCDs globally, with the death toll rising from 12.1 million in 1990 to 20.5 million in 2025, and possibly reaching 35.6 million by 2050 (1). CKD is another significant contributor to the global burden of NCDs. In 2019, it was estimated that 697 million individuals had CKD, resulting in 1.4 million deaths (27). Furthermore, the prevalence and death rate of diabetes worldwide are alarming, with 828 million cases and 1.4 million deaths reported in 2022 (28). Cardiovascular, kidney, and metabolic systems are intricately connected (29), with the onset of diseases in these areas often linked to common pathophysiological processes and risk factors, including dysglycemia, dyslipidemia, hypertension, and obesity (30). Therefore, dysfunction in one system can initiate and magnify dysfunction in others, affecting subsequent health outcomes and life expectancy (31, 32).

Considering the complex interplay of the mechanisms of the above conditions, the AHA proposed the concept of CKM syndrome staging in October 2023 (7). The staging incorporates abdominal obesity and other metabolic dysfunctions, as they may increase the risk of CKM syndrome progression. Individuals who do not present CKM health risk factors are classified as stage 0. Those with excess or dysfunctional adiposity are classified as stage 1, those with metabolic risk factors or moderate to high-risk CKD as stage 2, those with subclinical CVD or very high-risk CKD in CKM as stage 3, and those with clinical CVD in CKM syndrome as stage 4 (7). Recent study indicated that CKM syndrome is highly prevalent among U.S. adults, with about 90% meeting the criteria for CKM stage 1 or higher, and 15% of U.S. adults are identified as having advanced CKM stages (stage 3 or 4) (8). Moreover, based on a large, nationally representative cohort of U.S. adults, Zhu et al. found that compared with non-advanced CKM stages, the adjusted HR for all-cause premature mortality for advanced stages was 1.79 (1.53–2.10) (5). The poor CKM health highlights the urgent need for public health intervention to optimize the health of individuals in the U.S.

In 2022, the AHA introduced the LE8 score, a detailed and sensitive tool for assessing CVH (10). By comprehensively combining four modifiable health behaviors (diet, physical activity, nicotine exposure, sleep health) and four health factors (BMI, non-HDL cholesterol, blood glucose, blood pressure), the LE8 score is considered as an ideal CVH metric. Previous research involving different populations has indicated that achieving a high LE8 score could significantly reduce mortality (33–35). Besides, the LE8 score has demonstrated an inverse relationship with the prevalence of CVD (11), CKD (12), and diabetes (13). Given its close relationship to mortality and major CKM components, the LE8 score was hypothesized to be related to the risk of all-cause mortality in CKM syndrome patients, particularly in both advanced and non-advanced syndrome patients.

Interestingly, our analysis revealed a correlation between LE8 score and all-cause mortality in advanced CKM syndrome patients, when no correlation was found in non-advanced CKM syndrome patients. We consider that this nonsignificant may be attributed to four components of the LE8 score overlapping with the definition of CKM syndrome: BMI, blood lipid profile, blood glucose, and blood pressure. These shared factors may have attenuated the effect of LE8 score on the risk of all-cause mortality in non-advanced CKM syndrome patients. We then explored the association of the LE8 subscales, namely LE8 health behaviors and factors score, with all-cause mortality. As anticipated, the LE8 health behaviors score was significantly associated with all-cause mortality in both advanced and non-advanced CKM syndrome patients, while the LE8 health factors score failed to show the correlations. The RCS curve showed that a higher LE8 health behaviors score was associated with a largely reduced risk of all-cause mortality in a dose–response manner. For CKM syndrome patients, a small improvement in LE8 health behaviors score could lead to a large reduction in all-cause mortality, and this trend was more pronounced at lower LE8 health behaviors score, suggesting that CKM syndrome patients with lower LE8 health behaviors score may benefit more from a small improvement.

For now, this is the first study to evaluate the relationship between LE8 health behaviors score and all-cause mortality in CKM syndrome patients. The underlying potential mechanism may be related to insulin resistance. The AHA presidential advisory emphasizes that managing and preventing CKM syndrome primarily targets excess body fat and insulin resistance, which are key drivers of its harmful effects. Cardiovascular health behaviors, including a healthy diet, regular physical activity, and smoking cessation, are fundamental to the prevention of excessive weight gain and the improvement of insulin resistance (7). Social determinants of health (SDOH) might also be a main mechanism of this association. Unfavorable SDOHs (e.g., unemployed status, low food security, not married or living with a partner, etc.), were associated with higher odds of CKM multimorbidity (36) and increased risks of all-cause premature mortality across CKM stages (5). Adverse SDOHs have downstream consequences for events and mortality from CVD, CKD, and diabetes through behavioral pathways, such as physical inactivity and unhealthy diets (7, 37, 38).

Strengths of the study include the nationally representative sample of U.S. adults with CKM syndrome. In addition, we explored the associations between total LE8 score and its subscales (LE8 health behaviors and factors score) with mortality risk, providing convincing evidence that the LE8 health behaviors score is useful for assessing the risk of all-cause mortality in both advanced and non-advanced CKM syndrome patients. However, there are several potential limitations that need to be acknowledged. First, the observational study design prevented us from concluding a causal association between LE8 health behaviors score and mortality. Second, the assessment of LE8 health behavior indicators was based on self-report questionnaires, which are susceptible to recall bias. For example, participants may overestimate or underestimate physical activity levels, and perceptions of physical activity intensity may vary from person to person. However, the subjective measures of physical activity have been shown to correlate significantly with objective measures, such as accelerometry (39). Third, data on LE8 metrics were only obtained at baseline. Thus, we were unable to consider the impact of long-term variations in these CVH metrics over the course of follow-up. Further studies are needed to determine the relationship between dynamics of these scores and health outcomes in CKM syndrome patients. Fourth, certain CVD-related data, like echocardiographic parameters and coronary angiography, which are used to define advanced CKM syndrome stages, were not available in the NHANES database, potentially causing an underestimation of advanced CKM syndrome. Fifth, there were relatively few deaths from CVD and cancer in each CVH group, so we did not concentrate on mortality from these causes. Finally, our study is limited to the U.S. population and therefore cannot be generalized to other countries or other ethnic groups.

## Conclusion

In conclusion, in this nationally representative sample of U.S. adults, our study revealed a significant association between the LE8 health behaviors score and all-cause mortality among both advanced and non-advanced CKM syndrome patients. The LE8 health behaviors score could yield crucial insights for improving risk management approaches for CKM syndrome in future research and clinical practice.

## Data Availability

The original contributions presented in the study are included in the article/Supplementary material, further inquiries can be directed to the corresponding author/s.

## Glossary

AHA: American Heart Association
AIC: Akaike information criterion
BMI: Body mass index
CI: Confidence interval
CKD: Chronic kidney disease
CKM: Cardiovascular-Kidney-Metabolic
CVD: Cardiovascular disease
CVH: Cardiovascular health
eGFR: estimated glomerular filtration rate
HbA1c: Hemoglobin A1c
HDL: high-density lipoprotein
HEI-2015: Healthy Eating Index-2015
HR: hazard ratios
ICD-10: International Statistical Classification of Disease, Tenth Revision
LDL: low-density lipoprotein
LE8: Life’s Essential 8
NCDs: Non-communicable diseases
NHANES: National Health and Nutrition Examination Survey
non-HDL: non-high-density lipoprotein
PAF: population attributable fraction
PREVENT: Predicting Risk of CVD EVENTs
RCS: restricted cubic spline
SDOH: Social determinants of health
SE: Standard errors
STROBE: Strengthening the Reporting of Observational Studies in Epidemiology
