# Supplementary material for: Association between the life’s essential 8 health behaviors score and all-cause mortality in cardiovascular-kidney-metabolic syndrome patients
Source: Front Nutr. 2025 Jun 27;12:1612693. doi: 10.3389/fnut.2025.1612693 (PMC12245692; doi:10.3389/fnut.2025.1612693)
Supplement: Supplementary file 1 [file Table_1.DOCX]

**Table S1.** Definition and scoring approach for the Life’s Essential 8 score

| Domain | CVH Metric | Measurement | Quantification and Scoring of CVH Metric |
| --- | --- | --- | --- |
| Health Behaviors | Diet | Healthy Eating Index-2015 diet score percentile | **Metric:** Quantiles of Healthy Eating Index-2015  **Scoring:**  Points Quantile  100 ≥95th percentile (top/ideal diet)  80 75th – 94th percentile  50 50th – 74th percentile  25 25th – 49th percentile  0 1st – 24th percentile (bottom/least ideal quartile) |
|  | Physical activity | Self-reported minutes of moderate or vigorous physical activity per week | **Metric:** Minutes of moderate or greater intensity activity per week  **Scoring:**  Points Minutes  100 ≥150  90 120 – 149  80 90 – 119  60 60 – 89  40 30 – 59  20 1 – 29  0 0 |
|  | Nicotine exposure | Self-reported use of cigarettes or inhaled nicotine-delivery system (NDS) | **Metric:** Combustible tobacco use or inhaled nicotine-delivery system use; or secondhand smoke exposure  **Scoring:**  Points Status  100 Never smoker  75 Former smoker, quit ≥5 years  50 Former smoker, quit 1 - <5 years  25 Former smoker, quit <1 year, or currently using inhaled NDS  0 Current smoker  Subtract 20 points (unless score is 0) for living with active indoor smoker in home |
|  | Sleep health | Self-reported average hours of sleep per night | **Metric:** Average hours of sleep per night  **Scoring:**  Points Level  100 7 – <9  90 9 – <10  70 6 – <7  40 5 – <6 or ≥10  20 4 – <5  0 <4 |
| Health Factors | Body mass index | Body weight (kilograms) divided by height squared (meters squared) | **Metric:** Body mass index (kg/m^2^)  **Scoring:**  Points Level  100 <25  70 25.0 – 29.9  30 30.0 – 34.9  15 35.0 – 39.9  0 ≥40.0 |
|  | Blood lipids | Plasma total and high-density lipoprotein (HDL)-cholesterol with calculation of non-HDL-cholesterol | **Metric:** Non-HDL-cholesterol (mg/dL)  **Scoring:**  Points Level  100 <130  60 130 – 159  40 160 – 189  20 190 – 219  0 ≥220  If drug-treated level, subtract 20 points |
|  | Blood glucose | Fasting blood glucose (FBG) or casual hemoglobin A1c (HbA1c) | **Metric:** FBG (mg/dL) or HbA1c (%)  **Scoring:**  Points Level  100 No history of diabetes and FBG <100 (or HbA1c < 5.7)  60 No diabetes and FBG 100 – 125 (or HbA1c 5.7-6.4) (Pre-diabetes)  40 Diabetes with HbA1c <7.0  30 Diabetes with HbA1c 7.0 – 7.9  20 Diabetes with HbA1c 8.0 – 8.9  10 Diabetes with Hb A1c 9.0 – 9.9  0 Diabetes with HbA1c ≥10.0 |
|  | Blood pressure | Appropriately measured systolic and diastolic blood pressure | **Metric:** Systolic and diastolic blood pressure (mm Hg)  **Scoring:**  Points Level  100 <120/<80 (Optimal)  75 120-129/<80 (Elevated)  50 130-139 or 80-89 (Stage I hypertension)  25 140-159 or 90-99  0 ≥160 or ≥100  Subtract 20 points if treated level |

Abbreviations: CVH, cardiovascular health; FBG, Fasting blood glucose; HbA1c, hemoglobin A1c; HDL, high-density lipoprotein; NDS, nicotine-delivery system.

**Table S2.** KDIGO risk for CKD classification

| **eGFR, ml/min per 1.73 m2** | **Urinary ACR, mg/g** | | |
| --- | --- | --- | --- |
|  | **<30** | **30 to 299** | **≥300** |
| **≥90** | low risk | moderate risk | high risk |
| **60-89** | low risk | moderate risk | high risk |
| **45-59** | moderate risk | high risk | very high risk |
| **30-44** | high risk | very high risk | very high risk |
| **15-29** | very high risk | very high risk | very high risk |
| **<15** | very high risk | very high risk | very high risk |

Abbreviations: ACR, albumin-creatinine ratio; CKD, chronic kidney disease; eGFR, estimated glomerular filtration rate; KDIGO, Kidney Disease Improving Global Outcomes.

**Table S3.** Definitions of CKM Syndrome Stages

| **CKM** **syndrome stages** | **Definition** |
| --- | --- |
| Stage 0 | Normal BMI (<25 kg/m2 or <23 kg/m2 if Asian ethnicity);  Normal waist circumference (<88/102 cm in women/men or <80/90 cm in women/men if Asian ethnicity);  Normoglycemia;  Normotension;  Normal lipid status;  No evidence of CKD;  No evidence of CVD. |
| Stage 1 | Elevated BMI (≥25 kg/m2 or ≥23 kg/m2 if Asian ethnicity);  Elevated waist circumference (≥88/102 cm in women/men or ≥80/90 cm in women/men if Asian ethnicity);  Prediabetes (fasting blood glucose between 100 mg/dL and 126 mg/dL or glycated hemoglobin between 5.7% and 6.4%). |
| Stage 2 | Elevated fasting serum triglycerides (≥135 mg/dL);  Hypertension (blood pressure ≥140/90 mmHg, self-reported history of hypertension, or current antihypertensive treatment);  Diabetes (fasting blood glucose ≥126 mg/dL, glycated hemoglobin≥6.5%, self-reported history of diabetes, or current hypoglycemic treatment);  Metabolic syndrome (≥3 of the following: elevated waist circumference, high density lipoprotein cholesterol <40 mg/dL for men and <50 mg/dL for women, fasting serum triglycerides ≥150 mg/dL, blood pressure ≥130/80 mmHg and/or current antihypertensive treatment, fasting blood glucose ≥100 mg/dL);  Moderate-to-high-risk CKD^1^. |
| Stage 3 | Very-high-risk CKD^1^;  High predicted 10-year CVD risk^2^. |
| Stage 4 | Self-reported established CVD (coronary heart disease, angina, heart attack, heart failure, and stroke). |

^1^CKD stages were identified based on GFR and urinary albumin-to creatinine ratio (Table S2).

^2^The 10-year CVD risk was calculated using the American Heart Association Predicting Risk of CVD EVENTs equations.

Abbreviations: BMI, Body Mass Index; CKD, chronic kidney disease; CKM, Cardiovascular-Kidney-Metabolic; CVD, cardiovascular disease.

**Table S4.** The tests for Cox proportional hazards assumption using Schoenfeld residuals

| **Weighted Cox model** | *P* for global Schoenfeld test | |  | |
| --- | --- | --- | --- | --- |
|  | Model 1 | Model 2 |  | |
| **Categorical LE8 score** |  |  | |  |
| Overall | >0.05 | >0.05 | |  |
| Advanced CKM syndrome | >0.05 | >0.05 | |  |
| Non-advanced CKM syndrome | >0.05 | >0.05 | |  |
| **Categorical LE8 behavior score** |  |  | |  |
| Overall | >0.05 | >0.05 | |  |
| Advanced CKM syndrome | >0.05 | >0.05 | |  |
| Non-advanced CKM syndrome | >0.05 | >0.05 | |  |
| **Categorical LE8 factor score** |  |  | |  |
| Overall | >0.05 | >0.05 | |  |
| Advanced CKM syndrome | >0.05 | >0.05 | |  |
| Non-advanced CKM syndrome | >0.05 | >0.05 | |  |

Model 1: adjusted for sociodemographic variables (age, sex, ethnicity, educational level, family income, and marital status).

Model 2: Model 1 + body mass index, hemoglobin A1c, total cholesterol, high-density lipoprotein cholesterol, low-density lipoprotein cholesterol, estimated glomerular filtration rate, alcohol status, history of cancer, and the use of antihypertensive, antidiabetic, lipid-lowering medications.

The Schoenfeld Residuals Test demonstrated that proportional hazards assumption was met (all P for global Schoenfeld test >0.05).

Abbreviations: CI, confidence intervals; CKM, cardiovascular-kidney-metabolic; HR, hazard ratio; LE8, Life’s Essential 8.

**Table S5.** The multivariable-adjusted RCS for association of LE8 score with all-cause mortality: Selecting specific number of knots with the lowest AIC criterion value

| **Knots** | **AIC** | ***P* for non-linear** |
| --- | --- | --- |
| 3 | 11256.45 | 0.314 |
| 4 | 11258.48 | 0.611 |
| 5 | 11259.29 | 0.532 |
| 6 | 11257.91 | 0.224 |
| 7 | 11260.42 | 0.403 |
| 8 | 11262.05 | 0.478 |

Abbreviations: AIC, Akaike information criterion; RCS, restricted cubic spline.

**Table S6.** The multivariable-adjusted RCS for association of LE8 health behaviors score with all-cause mortality: Selecting specific number of knots with the lowest AIC criterion value

| **Knots** | **AIC** | ***P* for non-linear** |
| --- | --- | --- |
| 3 | 10279.18 | 0.280 |
| 4 | 10280.48 | 0.401 |
| 5 | 10281.44 | 0.402 |
| 6 | 10282.13 | 0.374 |
| 7 | 10284.62 | 0.589 |
| 8 | 10285.55 | 0.559 |

Abbreviations: AIC, Akaike information criterion; RCS, restricted cubic spline.

**Table S7.** The multivariable-adjusted RCS for association of LE8 health factors score with all-cause mortality: Selecting specific number of knots with the lowest AIC criterion value

| **Knots** | **AIC** | ***P* for non-linear** |
| --- | --- | --- |
| 3 | 10370.87 | <0.001 |
| 4 | 10369.43 | <0.001 |
| 5 | 10367.61 | <0.001 |
| 6 | 10367.34 | <0.001 |
| 7 | 10368.76 | <0.001 |
| 8 | 10370.89 | <0.001 |

Abbreviations: AIC, Akaike information criterion; RCS, restricted cubic spline.

**Table S8.** Variables-stratified analyses for the association between LE8 health behaviors score all-cause mortality in CKM syndrome patients

| **Characteristic** |  | **Health behaviors score** | | |  | ***P interaction*** | |
| --- | --- | --- | --- | --- | --- | --- | --- |
|  | **Low (0-49)** | **Moderate (50-79)** | | | **High (80-100)** |  |  |
| **Age** |  |  | | |  | 0.066 | |
| 20-44 | 1 (reference) | 0.76(0.38,1.50) | | | 0.26(0.07,0.97) |  | |
| 45-64 | 1 (reference) | 0.45(0.31,0.65) | | | 0.22(0.10,0.49) |  | |
| ≥65 | 1 (reference) | 0.65(0.51,0.82) | | | 0.37(0.26,0.53) |  | |
| **Sex** |  | |  | 0.053 | | |  |
| Female | 1 (reference) | 0.64(0.52,0.79) | | | 0.26(0.16,0.40) |  | |
| Male | 1 (reference) | 0.53(0.39,0.74) | | | 0.39(0.27,0.55) |  | |
| **Race or ethnicity** |  | |  | 0.203 | | |  |
| White | 1 (reference) | 0.53(0.43,0.65) | | | 0.31(0.22,0.42) |  | |
| Nonwhite | 1 (reference) | 0.79(0.57,1.10) | | | 0.39(0.26,0.58) |  | |
| **Educational level** |  | |  | 0.602 | | |  |
| Less than high school | 1 (reference) | 0.37(0.27,0.51) | | | 0.21(0.12,0.38) |  | |
| High school or equivalent | 1 (reference) | 0.65(0.45,0.96) | | | 0.61(0.34,0.97) |  | |
| College or above | 1 (reference) | 0.71(0.52,0.97) | | | 0.31(0.22,0.44) |  | |
| **Marital status** | 0.083 | | | | | |  |
| Married | 1 (reference) | 0.53(0.37,0.74) | | | 0.37(0.25,0.55) |  | |
| Single or separated | 1 (reference) | 0.69(0.54,0.89) | | | 0.29(0.17,0.47) |  | |
| **PIR** |  | |  | 0.124 | | |  |
| <1 | 1 (reference) | 0.81(0.60,1.11) | | | 0.27(0.14,0.54) |  | |
| 1-3 | 1 (reference) | 0.61(0.48,0.79) | | | 0.37(0.23,0.60) |  | |
| >3 | 1 (reference) | 0.46(0.30,0.71) | | | 0.28(0.16,0.48) |  | |
| **Alcohol status** |  |  | | |  | 0.375 | |
| Non-drinker | 1 (reference) | 0.57(0.44,0.74) | | | 0.26(0.18,0.37) |  | |
| Drinker | 1 (reference) | 0.59(0.48,0.74) | | | 0.38(0.26,0.55) |  | |
| **History of cancer** |  |  | | |  | 0.260 | |
| Yes | 1 (reference) | 0.68(0.47,0.97) | | | 0.48(0.29,0.79) |  | |
| No | 1 (reference) | 0.55(0.44,0.69) | | | 0.29(0.19,0.42) |  | |
| **Antihypertensive drugs** |  |  | | |  | 0.813 | |
| Yes | 1 (reference) | 0.59(0.49,0.70) | | | 0.32(0.23,0.46) |  | |
| No | 1 (reference) | 0.60(0.41,0.89) | | | 0.34(0.20,0.57) |  | |
| **Antidiabetic drugs** |  |  | | |  | 0.061 | |
| Yes | 1 (reference) | 0.48(0.33,0.70) | | | 0.41(0.22,0.77) |  | |
| No | 1 (reference) | 0.63(0.52,0.77) | | | 0.32(0.24,0.44) |  | |
| **Antihyperlipidemic drugs** |  |  | | |  | 0.253 | |
| Yes | 1 (reference) | 0.61(0.45,0.82) | | | 0.42(0.30,0.61) |  | |
| No | 1 (reference) | 0.56(0.44,0.70) | | | 0.26(0.19,0.37) |  | |

Values are weighted hazard ratios (95% confidence interval).

Model: each stratification was adjusted for age, sex, ethnicity, education level, marital status, body mass index, hemoglobin A1c, total cholesterol, high-density lipoprotein cholesterol, low-density lipoprotein cholesterol, estimated glomerular filtration rate, alcohol status, history of cancer, and the use of antihypertensive, antidiabetic, lipid-lowering medications, except the stratification factor itself.

Abbreviations: CKM, Cardiovascular-Kidney-Metabolic; PIR, poverty-to-income ratio.

**Table S9.** Variables-stratified analyses for the association between LE8 health behaviors score all-cause mortality in advanced CKM syndrome patients

| **Characteristic** |  | **Health behaviors score** | | |  | ***P interaction*** | |
| --- | --- | --- | --- | --- | --- | --- | --- |
|  | **Low (0-49)** | **Moderate (50-79)** | | | **High (80-100)** |  |  |
| **Age** |  |  | | |  | 0.276 | |
| 20-44 | 1 (reference) | 0.73(0.12,4.41) | | | 0.59(0.14,0.85) |  | |
| 45-64 | 1 (reference) | 0.62(0.34,1.12) | | | 0.12(0.02,0.64) |  | |
| ≥65 | 1 (reference) | 0.52(0.36,0.74) | | | 0.40(0.27,0.59) |  | |
| **Sex** |  | |  | 0.765 | | |  |
| Female | 1 (reference) | 0.52(0.37,0.72) | | | 0.30(0.16,0.57) |  | |
| Male | 1 (reference) | 0.52(0.32,0.85) | | | 0.36(0.21,0.61) |  | |
| **Race or ethnicity** |  | |  | 0.362 | | |  |
| White | 1 (reference) | 0.47(0.33,0.66) | | | 0.34(0.23,0.49) |  | |
| Nonwhite | 1 (reference) | 0.86(0.52,1.40) | | | 0.23(0.09,0.57) |  | |
| **Educational level** |  | |  | 0.054 | | |  |
| Less than high school | 1 (reference) | 0.37(0.20,0.68) | | | 0.26(0.12,0.56) |  | |
| High school or equivalent | 1 (reference) | 0.48(0.29,0.81) | | | 0.57(0.24,1.37) |  | |
| College or above | 1 (reference) | 0.59(0.39,0.90) | | | 0.29(0.18,0.46) |  | |
| **Marital status** | 0.818 | | | | | |  |
| Married | 1 (reference) | 0.55(0.34,0.90) | | | 0.42(0.25,0.71) |  | |
| Single or separated | 1 (reference) | 0.54(0.37,0.77) | | | 0.31(0.17,0.56) |  | |
| **PIR** |  | |  | 0.298 | | |  |
| <1 | 1 (reference) | 0.39(0.24,0.65) | | | 0.20(0.07,0.57) |  | |
| 1-3 | 1 (reference) | 0.58(0.41,0.81) | | | 0.42(0.21,0.82) |  | |
| >3 | 1 (reference) | 0.46(0.25,0.85) | | | 0.32(0.15,0.65) |  | |
| **Alcohol status** |  |  | | |  | 0.190 | |
| Non-drinker | 1 (reference) | 0.50(0.33,0.77) | | | 0.22(0.12,0.41) |  | |
| Drinker | 1 (reference) | 0.50(0.32,0.77) | | | 0.43(0.26,0.73) |  | |
| **History of cancer** |  |  | | |  | 0.184 | |
| Yes | 1 (reference) | 0.56(0.35,0.88) | | | 0.54(0.30,0.99) |  | |
| No | 1 (reference) | 0.48(0.35,0.66) | | | 0.25(0.15,0.43) |  | |
| **Antihypertensive drugs** |  |  | | |  | 0.053 | |
| Yes | 1 (reference) | 0.57(0.42,0.76) | | | 0.33(0.21,0.51) |  | |
| No | 1 (reference) | 0.25(0.11,0.59) | | | 0.29(0.12,0.69) |  | |
| **Antidiabetic drugs** |  |  | | |  | 0.496 | |
| Yes | 1 (reference) | 0.47(0.31,0.72) | | | 0.42(0.20,0.88) |  | |
| No | 1 (reference) | 0.55(0.38,0.79) | | | 0.32(0.22,0.47) |  | |
| **Antihyperlipidemic drugs** |  |  | | |  | 0.207 | |
| Yes | 1 (reference) | 0.62(0.41,0.96) | | | 0.41(0.26,0.67) |  | |
| No | 1 (reference) | 0.38(0.21,0.68) | | | 0.29(0.17,0.50) |  | |

Values are weighted hazard ratios (95% confidence interval).

Model: each stratification was adjusted for age, sex, ethnicity, education level, marital status, body mass index, hemoglobin A1c, total cholesterol, high-density lipoprotein cholesterol, low-density lipoprotein cholesterol, estimated glomerular filtration rate, alcohol status, history of cancer, and the use of antihypertensive, antidiabetic, lipid-lowering medications, except the stratification factor itself.

Abbreviations: CKM, Cardiovascular-Kidney-Metabolic; PIR, poverty-to-income ratio.

**Table S10.** Variables-stratified analyses for the association between LE8 health behaviors score all-cause mortality in non-advanced CKM syndrome patients

| **Characteristic** |  | **Health behaviors score** | | |  | ***P interaction*** | |
| --- | --- | --- | --- | --- | --- | --- | --- |
|  | **Low (0-49)** | **Moderate (50-79)** | | | **High (80-100)** |  |  |
| **Age** |  |  | | |  | 0.079 | |
| 20-44 | 1 (reference) | 0.82(0.39,1.71) | | | 0.29(0.07,1.14) |  | |
| 45-64 | 1 (reference) | 0.52(0.29,0.93) | | | 0.32(0.13,0.79) |  | |
| ≥65 | 1 (reference) | 1.08(0.72,1.63) | | | 0.45(0.25,0.80) |  | |
| **Sex** |  | |  | 0.350 | | |  |
| Female | 1 (reference) | 0.95(0.66,1.38) | | | 0.31(0.18,0.54) |  | |
| Male | 1 (reference) | 0.60(0.38,0.93) | | | 0.45(0.25,0.82) |  | |
| **Race or ethnicity** |  | |  | 0.720 | | |  |
| White | 1 (reference) | 0.70(0.47,1.06) | | | 0.34(0.19,0.60) |  | |
| Nonwhite | 1 (reference) | 0.77(0.51,1.16) | | | 0.48(0.31,0.74) |  | |
| **Educational level** |  | |  | 0.159 | | |  |
| Less than high school | 1 (reference) | 0.36(0.19,0.70) | | | 0.16(0.06,0.40) |  | |
| High school or equivalent | 1 (reference) | 1.03(0.51,2.08) | | | 0.80(0.34,1.86) |  | |
| College or above | 1 (reference) | 1.08(0.66,1.77) | | | 0.44(0.26,0.76) |  | |
| **Marital status** | 0.526 | | | | | |  |
| Married | 1 (reference) | 0.53(0.33,0.86) | | | 0.37(0.20,0.68) |  | |
| Single or separated | 1 (reference) | 1.19(0.84,1.70) | | | 0.38(0.20,0.70) |  | |
| **PIR** |  | |  | 0.146 | | |  |
| <1 | 1 (reference) | 1.32(0.72,2.43) | | | 0.44(0.18,1.08) |  | |
| 1-3 | 1 (reference) | 0.76(0.52,1.11) | | | 0.40(0.24,0.67) |  | |
| >3 | 1 (reference) | 0.49(0.27,0.91) | | | 0.28(0.11,0.69) |  | |
| **Alcohol status** |  |  | | |  | 0.958 | |
| Non-drinker | 1 (reference) | 0.78(0.47,1.31) | | | 0.37(0.20,0.69) |  | |
| Drinker | 1 (reference) | 0.75(0.53,1.05) | | | 0.39(0.23,0.67) |  | |
| **History of cancer** |  |  | | |  | 0.353 | |
| Yes | 1 (reference) | 1.05(0.50,2.19) | | | 0.46(0.18,1.19) |  | |
| No | 1 (reference) | 0.67(0.46,0.98) | | | 0.36(0.22,0.61) |  | |
| **Antihypertensive drugs** |  |  | | |  | 0.619 | |
| Yes | 1 (reference) | 0.77(0.52,1.16) | | | 0.40(0.21,0.77) |  | |
| No | 1 (reference) | 0.77(0.49,1.19) | | | 0.36(0.19,0.69) |  | |
| **Antidiabetic drugs** |  |  | | |  | 0.373 | |
| Yes | 1 (reference) | 0.70(0.36,1.39) | | | 0.61(0.18,2.06) |  | |
| No | 1 (reference) | 0.74(0.54,1.02) | | | 0.36(0.23,0.57) |  | |
| **Antihyperlipidemic drugs** |  |  | | |  | 0.361 | |
| Yes | 1 (reference) | 1.02(0.53,2.00) | | | 0.74(0.31,1.75) |  | |
| No | 1 (reference) | 0.70(0.50,0.97) | | | 0.29(0.18,0.45) |  | |

Values are weighted hazard ratios (95% confidence interval).

Model: each stratification was adjusted for age, sex, ethnicity, education level, marital status, body mass index, hemoglobin A1c, total cholesterol, high-density lipoprotein cholesterol, low-density lipoprotein cholesterol, estimated glomerular filtration rate, alcohol status, history of cancer, and the use of antihypertensive, antidiabetic, lipid-lowering medications, except the stratification factor itself.

Abbreviations: CKM, Cardiovascular-Kidney-Metabolic; PIR, poverty-to-income ratio.

**Table S11.** Association between LE8 health behaviors score and all-cause mortality in CKM syndrome patients after excluding death within the first year of follow-up

| **Health behaviors score** | Model 1 | |  | Model 2 | |
| --- | --- | --- | --- | --- | --- |
|  | HR (95% CI) | *P* value |  | HR (95% CI) | *P* value |
| **Overall** |  |  | |  |  |
| Low (0-49) | 1 (reference) |  | | 1 (reference) |  |
| Moderate (50-79) | 0.62(0.52,0.75) | <0.0001 | | 0.64(0.53,0.77) | <0.0001 |
| High (80-100) | 0.33(0.24,0.45) | <0.0001 | | 0.35(0.26,0.47) | <0.0001 |
| Per 10 score increase | 0.81(0.78,0.84) | <0.0001 | | 0.82(0.78,0.85) | <0.0001 |
| **Advanced CKM syndrome** |  |  | |  |  |
| Low (0-49) | 1 (reference) |  | | 1 (reference) |  |
| Moderate (50-79) | 0.57(0.43,0.75) | <0.0001 | | 0.58(0.44,0.77) | <0.0001 |
| High (80-100) | 0.34(0.23,0.51) | <0.0001 | | 0.36(0.25,0.53) | <0.0001 |
| Per 10 score increase | 0.79(0.74,0.84) | <0.0001 | | 0.79(0.75,0.84) | <0.0001 |
| **Non-advanced CKM syndrome** |  |  | |  |  |
| Low (0-49) | 1 (reference) |  | | 1 (reference) |  |
| Moderate (50-79) | 0.79(0.56,1.11) | <0.0001 | | 0.74(0.56,0.98) | 0.04 |
| High (80-100) | 0.39(0.24,0.62) | <0.0001 | | 0.41(0.25,0.66) | <0.001 |
| Per 10 score increase | 0.85(0.79,0.92) | <0.0001 | | 0.86(0.79,0.93) | <0.001 |

Model 1: adjusted for sociodemographic variables (age, sex, ethnicity, educational level, family income, and marital status).

Model 2: Model 1 + body mass index, hemoglobin A1c, total cholesterol, high-density lipoprotein cholesterol, low-density lipoprotein cholesterol, estimated glomerular filtration rate, alcohol status, history of cancer, and the use of antihypertensive, antidiabetic, lipid-lowering medications.

Abbreviations: CI, confidence intervals; CKM, cardiovascular-kidney-metabolic; HR, hazard ratio; LE8, Life’s Essential 8.

**Table S12.** Association between LE8 health behaviors score and all-cause mortality in CKM syndrome patients after excluding individuals with history of cancer

| **Health behaviors score** | Model 1 | |  | Model 2 | |
| --- | --- | --- | --- | --- | --- |
|  | HR (95% CI) | *P* value |  | HR (95% CI) | *P* value |
| **Overall** |  |  | |  |  |
| Low (0-49) | 1 (reference) |  | | 1 (reference) |  |
| Moderate (50-79) | 0.54(0.44,0.68) | <0.0001 | | 0.55(0.44,0.69) | <0.0001 |
| High (80-100) | 0.28(0.19,0.41) | <0.0001 | | 0.29(0.19,0.42) | <0.0001 |
| Per 10 score increase | 0.79(0.75,0.84) | <0.0001 | | 0.80(0.75,0.85) | <0.0001 |
| **Advanced CKM syndrome** |  |  | |  |  |
| Low (0-49) | 1 (reference) |  | | 1 (reference) |  |
| Moderate (50-79) | 0.48(0.36,0.65) | <0.0001 | | 0.48(0.35,0.66) | <0.0001 |
| High (80-100) | 0.25(0.15,0.41) | <0.0001 | | 0.25(0.15,0.43) | <0.0001 |
| Per 10 score increase | 0.76(0.71,0.82) | <0.0001 | | 0.76(0.70,0.82) | <0.0001 |
| **Non-advanced CKM syndrome** |  |  | |  |  |
| Low (0-49) | 1 (reference) |  | | 1 (reference) |  |
| Moderate (50-79) | 0.67(0.46,0.96) | 0.03 | | 0.67(0.46,0.98) | 0.04 |
| High (80-100) | 0.36(0.22,0.61) | <0.001 | | 0.36(0.22,0.61) | <0.001 |
| Per 10 score increase | 0.84(0.76,0.91) | <0.0001 | | 0.84(0.77,0.92) | <0.0001 |

Model 1: adjusted for sociodemographic variables (age, sex, ethnicity, educational level, family income, and marital status).

Model 2: Model 1 + body mass index, hemoglobin A1c, total cholesterol, high-density lipoprotein cholesterol, low-density lipoprotein cholesterol, estimated glomerular filtration rate, alcohol status, history of cancer, and the use of antihypertensive, antidiabetic, lipid-lowering medications.

Abbreviations: CI, confidence intervals; CKM, cardiovascular-kidney-metabolic; HR, hazard ratio; LE8, Life’s Essential 8.

**Table S13.** Association between LE8 health behaviors score and all-cause mortality in CKM syndrome patients after iterative imputation for missing data on covariates

| **Health behaviors score** | Model 1 | |  | Model 2 | |
| --- | --- | --- | --- | --- | --- |
|  | HR (95% CI) | *P* value |  | HR (95% CI) | *P* value |
| **Overall** |  |  | |  |  |
| Low (0-49) | 1 (reference) |  | | 1 (reference) |  |
| Moderate (50-79) | 0.60(0.50,0.71) | <0.0001 | | 0.60(0.49,0.72) | <0.0001 |
| High (80-100) | 0.35(0.27,0.45) | <0.0001 | | 0.36(0.29,0.46) | <0.0001 |
| Per 10 score increase | 0.81(0.78,0.84) | <0.0001 | | 0.81(0.78,0.84) | <0.0001 |
| **Advanced CKM syndrome** |  |  | |  |  |
| Low (0-49) | 1 (reference) |  | | 1 (reference) |  |
| Moderate (50-79) | 0.54(0.41,0.71) | <0.0001 | | 0.53(0.40,0.71) | <0.0001 |
| High (80-100) | 0.33(0.22,0.48) | <0.0001 | | 0.35(0.25,0.49) | <0.0001 |
| Per 10 score increase | 0.79(0.74,0.84) | <0.0001 | | 0.79(0.74,0.84) | <0.0001 |
| **Non-advanced CKM syndrome** |  |  | |  |  |
| Low (0-49) | 1 (reference) |  | | 1 (reference) |  |
| Moderate (50-79) | 0.73(0.55,0.97) | 0.03 | | 0.73(0.55,0.99) | 0.04 |
| High (80-100) | 0.43(0.30,0.60) | <0.0001 | | 0.45(0.32,0.63) | <0.0001 |
| Per 10 score increase | 0.85(0.80,0.90) | <0.0001 | | 0.85(0.80,0.90) | <0.0001 |

Model 1: adjusted for sociodemographic variables (age, sex, ethnicity, educational level, family income, and marital status).

Model 2: Model 1 + body mass index, hemoglobin A1c, total cholesterol, high-density lipoprotein cholesterol, low-density lipoprotein cholesterol, estimated glomerular filtration rate, alcohol status, history of cancer, and the use of antihypertensive, antidiabetic, lipid-lowering medications.

Abbreviations: CI, confidence intervals; CKM, cardiovascular-kidney-metabolic; HR, hazard ratio; LE8, Life’s Essential 8.
